# Exploring the Prevalence of PTSD in Hand Trauma: A Comprehensive Study

**DOI:** 10.3390/brainsci13101438

**Published:** 2023-10-10

**Authors:** Alexandra Florinda Ghițan, Veronica Gheorman, Marius Eugen Ciurea, Victor Gheorman, Venera Cristina Dinescu, Ana Maria Ciurea, Felicia Militaru, Romeo Popa, Tiberiu-Ștefăniță Țenea-Cojan, Ion Udriștoiu

**Affiliations:** 1 Doctoral School, University of Medicine and Pharmacy of Craiova, 200349 Craiova, Romania; ghitanalexandra@gmail.com; 2 Department of Cardiology, University of Medicine and Pharmacy of Craiova, 200349 Craiova, Romania; 3 Department of Plastic Surgery, University of Medicine and Pharmacy of Craiova, 200349 Craiova, Romania; meciurea@gmail.com; 4 Department of Psychiatry, University of Medicine and Pharmacy of Craiova, 200349 Craiova, Romania; victor.gheorman@umfcv.ro (V.G.); feliciobanu@yahoo.com (F.M.); ion.udristoiu@umfcv.ro (I.U.); 5 Department of Health Promotion and Occupational Medicine, University of Medicine and Pharmacy of Craiova, 200349 Craiova, Romania; venera.dinescu@umfcv.ro; 6 Department of Oncology, University of Medicine and Pharmacy of Craiova, 200349 Craiova, Romania; amciurea14@gmail.com; 7 Department of Pharmacology, University of Medicine and Pharmacy of Craiova, 200349 Craiova, Romania; romeo_rop@yahoo.com; 8 Department of Surgery, University of Medicine and Pharmacy of Craiova, 200349 Craiova, Romania; doctorts@yahoo.com

**Keywords:** hand trauma, impact, PTSD scoring

## Abstract

Hand trauma is a common and debilitating condition that can have significant physical, functional, and psychological effects on individuals. This study used a case–control design to investigate the frequency and factors associated with symptoms of post-traumatic stress disorder (PTSD) in a sample of individuals with complex hand and forearm injuries. Our hypothesis suggests that demographic data, among other factors, influences the intensity of PTSD symptoms measured by the PCL-5 scale three months post-surgery. This study included 166 individuals, 142 males and 24 females, with an average age of 42.14 years (SD = 12.71). Our study found significant associations between symptoms of PTSD and various demographic and clinical factors. PTSD symptoms were observed in females, individuals from specific regions, and certain socio-professional groups. Furthermore, educational attainment and personal background have been identified as significant factors in the development of PTSD. The role of trauma type was crucial, amputees and fractures were more prone to developing PTSD. A strong link was found between increased symptoms of PTSD and negative postoperative outcomes, including amputation of necessity and the need for additional surgery. The absence of family support exacerbates the psychological distress of trauma survivors. The findings highlight the intricate nature of PTSD development and underscore the significance of a comprehensive postoperative treatment strategy encompassing psychological assessment and support.

## 1. Introduction

Hand trauma refers to injuries sustained to the hand, including fractures, lacerations, amputations, and crush injuries. These traumatic events can have a significant impact on the lives of individuals, affecting their physical function, psychological well-being, and overall quality of life. Hand trauma often leads to functional limitations, chronic pain, and disability, necessitating comprehensive management strategies [1].

Based on a recent comprehensive examination of fracture and digit amputation cases as documented in the Global Burden of Disease 2017 Study, it has been observed that the worldwide occurrence of hand trauma has experienced a relatively minor decline from the year 1990 [2]. The age-standardised incidence rate of hand and wrist fractures in the year 2017 was recorded at 179 per 100,000 individuals [2].

The prevalence of work-related injuries with mangled hand lesions is significant in emergency rooms globally, whereas there is a dearth of studies on such injuries within the general population. Lacerations are the most often reported injuries on a global scale. Fractures, dislocations, ligamentous injuries, contusions, and amputations are among the frequently encountered types of lesions. According to a recent study, it was shown that those who were left-handed had a significantly higher likelihood, approximately 4.9-times greater, of experiencing a digit amputation compared to their right-handed counterparts [3].

The epidemiology of hand and wrist injuries in Romania lacks comprehensive documentation, both in the working and non-working populations. Additionally, there is a notable absence of a national registry specifically dedicated to recording hand injuries and the resulting disabilities. The prevalence of hand trauma in a specific geographic region like Oltenia in Romania could be influenced by a myriad of factors, such as the occupational landscape, socioeconomic status, and healthcare accessibility. Hand trauma represents a significant burden on healthcare systems worldwide and has a distinct epidemiological profile.

In conjunction with the physiological ramifications, hand trauma has the potential to engender substantial psychological and emotional distress. Patients may experience symptoms of anxiety, depression, and post-traumatic stress disorder (PTSD) following their injury. The psychological impact can arise from the traumatic event itself, the physical and functional changes resulting from the injury, and the challenges associated with the recovery process [4]. Psychological assessment and intervention are essential components of hand trauma management for addressing the emotional needs of patients.

PTSD is a psychiatric disorder that can develop in individuals who have experienced or witnessed a traumatic event, learnt about a trauma that occurred to a loved one, or were exposed to aversive details of trauma (typically in the course of professional duties). It is characterised by symptoms such as intrusive thoughts, nightmares, flashbacks, hypervigilance, and avoidance of trauma-related stimuli [5]. Recent research has recognised the prevalence of PTSD in individuals with hand trauma, with studies reporting rates ranging from 8% to 24% [6,7]. The presence of PTSD can significantly impact the cognitive, emotional, and psychosocial aspects of hand trauma patients. In a recent study, the prevalence of PTSD among hand trauma patients was reported to be 15%, highlighting the substantial burden of psychological trauma in this population [8].

The evidence underscores a significant correlation between the severity of hand trauma and increased PTSD incidence and symptom intensity. Studies indicate that patients with more severe injuries are at a heightened risk and exhibit exacerbated PTSD symptoms [8,9].

Understanding the prevalence of PTSD among hand trauma patients is critical for developing targeted treatment and rehabilitation strategies. Empirical findings attest to the efficacy of cognitive-behavioural therapy in mitigating PTSD symptoms [10]. Foundational insights from earlier studies underscore the necessity for embedding psychological support into comprehensive treatment frameworks to enhance overall patient outcomes [11].

New therapeutic approaches are emerging in the field of PTSD management, showing promise for improving patient outcomes. Oxytocin, a neuropeptide known for its involvement in social bonding and reproductive processes, shows promise in reducing the severity of PTSD symptoms. Recent studies have shown that it can help reduce hyperarousal and improve social cognition in individuals with PTSD, leading to better psychological well-being and resilience [12].

The use of MDMA-assisted therapy has revolutionised the treatment of severe PTSD. Clinical trials have demonstrated the effectiveness of this treatment in promoting significant psychological insights, emotional regulation, and reintegration, thereby enhancing the depth and effectiveness of the therapeutic process [13]. This is especially clear in cases where traditional therapies have not been effective, highlighting its potential as a key tool in managing PTSD.

Psilocybin and LSD have shown potential for significant psychological healing when administered in controlled and guided environments. Recent research supports the notion that they can aid in emotional release, cognitive flexibility, and the reconfiguration of traumatic memories, leading to a healing process that is not achievable through conventional methods [14].

Advancements in neuroimaging and neurofeedback techniques are leading to the development of customised, real-time interventions for highly individualised PTSD management. These innovations enable the direct modulation of brain activity patterns related to traumatic stress, providing a targeted intervention strategy that aligns with each patient’s specific neural and psychological characteristics [15].

We carefully identified potential predictors for our study, as recurrent themes highlighted key factors that worsen the development and severity of PTSD symptoms. Our goal was to create a detailed model that represents the complex nature of PTSD, with a focus on patients who have hand injuries.

While the mechanisms underlying the relationship between hand trauma and the onset of PTSD are complex and multifactorial, several factors have been identified. The physical trauma itself can be a traumatic event that triggers a stress response and subsequent development of PTSD symptoms. The loss of hand function and perceived disfigurement can also contribute to feelings of distress and loss of identity, which may exacerbate PTSD symptoms [16,17]. Additionally, preexisting psychological vulnerabilities, such as a history of trauma or mental health disorders, can influence an individual’s susceptibility to developing PTSD following hand trauma [18].

Gender differences in PTSD prevalence post-trauma have been extensively studied, with women often demonstrating a higher susceptibility to PTSD than men. Recent studies reiterate these findings, attributing the variance to differences in trauma type exposure, societal roles, and hormonal and biological distinctions [19].

The influence of age on PTSD has been intricately explored, younger age groups are more vulnerable to PTSD, attributable to factors like less developed coping mechanisms and resilience, variable trauma exposure, and ongoing neural maturation processes [20].

Environmental settings significantly influence PTSD incidence. Rural environments can exacerbate PTSD symptoms due to limited access to mental health services and increased stigma around mental health issues [21].

The relationship between socioeconomic status and PTSD is well-documented, with lower socioeconomic groups facing an amplified risk due to various stressors and limited access to quality healthcare [22].

The inclusion of standardised scoring for PTSD is an essential component within the comprehensive strategy to evaluate the initial and continuous psychological state of individuals who have suffered hand trauma. It is acknowledged that the comprehensive assessment should incorporate examinations for other commonly occurring mental health illnesses, such as depression and anxiety, and take into account the wider implications on patients’ general quality of life.

The integration of standardised PTSD scoring into the assessment and ongoing monitoring of hand trauma patients is instrumental for comprehensive care. It facilitates an enriched understanding of the patient’s mental health, enabling personalised treatment that encompasses both physical and psychological needs [23]. The research underscores a notable association between elevated PTSD symptoms and diminished functional outcomes in these patients, accentuating the imperative for early detection and intervention [24]. A multidisciplinary approach, integrating PTSD scoring, has been validated to enhance psychological well-being and expedite recovery, attesting to its pivotal role in optimising hand trauma management [25].

While the extant literature has indeed established a connection between hand trauma and subsequent PTSD, a nuanced understanding of this relationship remains largely unexplored. Our study aims to bridge this gap, offering an in-depth examination of the multifaceted interactions between diverse patient attributes, trauma categories, and the resultant PTSD severity.

We hypothesise that certain factors, such as being female, being younger, having lower educational attainment, and coming from a lower socioeconomic background, may contribute to the manifestation of symptoms of PTSD, which is consistent with prior research on PTSD. Furthermore, we suggest that the severity of trauma and the laterality of the affected hand are important factors in determining the extent of PTSD symptoms. Our hypothesis also considers the potential impact of family support in the social context; we expect to find a negative relationship between increased family support and decreased severity of PTSD symptoms, indicating the protective influence of social support as shown in previous research. After surgery, we believe there is a connection between the quality of surgical outcomes and the severity of PTSD symptoms, hypothesising that better physical recovery is associated with less psychological distress, which is supported by previous clinical knowledge.

## 2. Materials and Methods

### 2.1. Participants

This case–control study aimed to assess the prevalence of post-traumatic stress disorder among individuals with hand trauma and examine its association with various demographic, injury-related, medical treatment, and postoperative factors. Data were collected from individuals treated at the Plastic Surgery, Reconstructive Microsurgery and Burns Clinic in the Emergency County Clinical Hospital Craiova, Romania between 1 November 2021 and 31 March 2023. Demographic variables (gender, age, environment (specifically Oltenia counties), education level, and occupation), injury-related variables (cause of hand trauma, circumstances, affected hand, and type of wound), medical treatment variables (presence of amputation, presence of fractures, osteosynthesis, vascular ligation, nerve repair, and tendon repair), and postoperative variables (postoperative progression, type of reconstruction, use of a cast, need for subsequent surgical intervention, impact of family support, and socio-professional reintegration) were analysed using descriptive statistics and inferential analyses.

In our study, we utilised inclusion and exclusion criteria to determine the eligibility of participants.

Inclusion Criteria:

1. Adult patients (18 years and above) with a documented history of hand trauma.
2. Patients diagnosed with or suspected to have hand trauma-related injuries, including fractures, dislocations, or soft tissue injuries.
3. Patients with sufficient cognitive and linguistic abilities to understand and respond to study assessments and questionnaires.
4. Patients willing to attend follow-up appointments and complete the required study procedures.

Exclusion Criteria:

1. Patients with pre-existing psychiatric conditions or a history of psychological disorders unrelated to hand trauma.
2. Patients with severe cognitive impairment or language barriers that hinder comprehension and participation in the study.
3. Patients with acute medical conditions or ongoing interventions that may confound assessment and management of hand trauma-related PTSD.
4. Patients over 70 years old, as this age group may have a higher risk of complications associated with surgical interventions or specific treatments for hand trauma.
5. Patients with severe or multiple chronic diseases, such as cardiac or respiratory conditions, that may increase the risk of surgery or impact rehabilitation and recovery after hand injuries.
6. Patients with progressive or degenerative neurological conditions that may hinder hand recovery following trauma.
7. Patients with severe haematologic or immunologic conditions that may interfere with the healing process and increase the risk of infectious complications after hand trauma.

The research investigated a representative sample of 166 participants, ranging in age from 18 to 70 years at the time of diagnosis. The patients were classified into three age groups: 19–29, 30–49, and 50–70 years old. Age-specific segmentation is crucial for understanding the complex dynamics and multiple impacts of PTSD.

The age group of 19–29 is characterised by distinct vulnerabilities and manifestations of PTSD in this demographic. The unique presentation of PTSD in young adults is well-documented and linked to the specific developmental and psychosocial changes that occur during this life stage. This classification allows for a more targeted analysis of the complex experiences and difficulties faced by individuals in this age group, leading to a deeper understanding of the effects of PTSD [19].

The middle adult cohort, aged 30–49, is defined in relation to existing literature that highlights the importance of established life roles and cumulative life experiences in influencing the development of PTSD. This age group is characterised by a combination of factors, such as increased resilience and vulnerabilities, which require a separate categorisation to understand the unique trajectories of PTSD and coping mechanisms [26].

The classification of individuals aged 50–70 is based on research showing the connections between PTSD and common chronic health conditions in older age. The worsening of PTSD symptoms, combined with the increasing occurrence of health issues, highlights the need for a specific study of this group [27].

### 2.2. Procedures

The study protocol was thoroughly reviewed and approved by the Ethics Committee to ensure compliance with the Helsinki Declaration of 1975. The ethical importance of voluntary participation was emphasised and informed consent was obtained from each participant prior to the commencement of the research.

The deliberate choice to exclude patients with a preexisting mental history was a strategic decision made with the intention of minimising the influence of extraneous factors. Our objective was to establish a direct link between the observed symptoms of PTSD and the traumatic hand episode, in order to facilitate a definitive and unambiguous interpretation of the collected data.

This study was conducted in two phases. At the initiation of our investigation, every patient, upon their admittance to the plastic surgery clinic, underwent a thorough evaluation conducted by a multidisciplinary team consisting of both a psychologist and a psychiatrist. The primary assessment played a crucial role in establishing the psychological and psychiatric foundation of every participant, guaranteeing a targeted and precise analysis of the developing symptoms of post-traumatic stress disorder following hand injuries.

During the three-month period subsequent to their discharge, participants were asked to fill out a questionnaire as part of the follow-up process. The substantial number of patients who withdrew from this study in the second phase, during the three-month follow-up period, constituted a significant obstacle. The drop observed in our study was related to the existing societal attitudes toward mental health within our specific geographical and cultural setting. The conspicuous stigmatisation linked to mental illness exerts a profound impact on individuals’ inclination to acknowledge and confront mental health concerns.

The surveys were administered to all participants with no time limit, ensuring that each individual could take the necessary time to reflect on each question for accurate responses. In cases where participants were unsure of how to respond, clarifications and assistance were provided to ensure that their answers were as precise and representative of their experiences as possible.

### 2.3. Measures

In the assessment of PTSD in individuals with hand trauma, we used standardised measures like the Post-traumatic Stress Disorder Checklist for DSM-5 (PCL-5), a validated self-report questionnaire [21]. By assessing four symptom clusters of PTSD-intrusion, avoidance, negative alterations in cognition and mood, and alterations in arousal and reactivity—the PCL-5 provides valuable insights into the severity of symptoms experienced.

The PCL-5, a 20-item questionnaire, evaluates PTSD symptoms in alignment with the DSM-5 criteria, divided into four symptom clusters. These clusters focus on intrusion symptoms, avoidance behaviours, negative alterations in cognition and mood, and alterations in arousal and reactivity. Each item is rated on a scale of 0 to 4, with higher scores indicating more severe PTSD symptoms. Two approaches were employed to establish a preliminary diagnosis of PTSD:

1. A cut-off raw score of 38. The cut-off point exhibits a notable level of sensitivity (0.78) and specificity (0.98) [28].
2. At least 1 symptom from the B item category (questions 1–5), at least 1 symptom from the C item category (questions 6–7), at least 2 symptoms from the D item category (questions 8–14), and at least 2 symptoms from the E item category (questions 15–20) [29].

To gather information on psychiatric disorders, we conducted diagnostic interviews and had the patients complete self-reported questionnaires, with the option for follow-up interviews to clarify responses. The interviews were conducted by psychiatrists. We also conducted interviews with patients and consulted medical records to obtain information on family history. A standardised family history questionnaire was used to collect standardised information on the mental health and medical history of the patient’s family. Medication use details were obtained through a review of medical records, medication lists provided by patients, and self-reported questionnaires. Medical conditions were evaluated based on self-reports from patients, a review of their medical records, and physical examinations.

### 2.4. Statistical Analyses

For statistical analysis, we employed tests including the *t*-test and chi-square test and Pearson correlation. The chi-square test helped us assess associations between variables, such as gender, age groups, environment, county, causality of hand trauma, type of injuries, hand affected, postoperative evolution, subsequent surgical intervention, and family support. The *t*-test correlation was used to examine the relationship between continuous variables, as the presence of PTSD and the number of tendons affected. These statistical analyses provided insights into the impact of different factors on PTSD in individuals with hand trauma.

The data analysis was conducted using Windows 10 (Version 20H2, Microsoft Corp., Redmond, WA, USA, 2020), and MS Office Excel 2016 (Version 16.0.4266, Microsoft Corp., Redmond, WA, USA, 2015). IBM SPSS Statistics (Version 29.0, SPSS Inc., IBM Corp., Chicago, IL, USA, 2021) was used for all statistical analyses. A significance level of *p* < 0.05 was considered statistically significant.

## 3. Results

In assessing PTSD symptom severity, the PTSD group, comprised 86 individuals, exhibited a mean PCL-5 score of 31.4 (±6.35). In contrast, the non-PTSD group, consisting of 80 participants, recorded a mean PCL-5 score of 12.95 (±4.38). This clear discrepancy underscores the pronounced variation in symptom severity between the two groups, aligning with our study’s objective to delineate these differential presentations post-trauma.

### 3.1. Patient Characteristics

Several demographic characteristics of the patients were evaluated (Table 1). In our context of hand trauma, we observed a noticeable variation in PTSD prevalence across genders. Among the patients with PTSD symptoms, 19 were female and 67 were male; conversely, in the non-PTSD group, only 5 were female and 75 were male. A chi-square test rendered a *p*-value of 0.004, illustrating a significant gender disparity. This suggests that within the specific confines of hand trauma, gender may wield a significant influence on PTSD emergence and progression.

Our analysis of the age distribution among patients with and without PTSD revealed no statistically significant difference (*p* > 0.05). Patients with PTSD had a mean age of 45.17 years (SD = 15.24), while those without PTSD averaged at 42.14 years (SD = 12.71). The categorisation into age groups 18–29, 30–49, and 50–70 yielded comparable distributions across both PTSD and non-PTSD cohorts. A post-hoc power analysis, employing a Cohen’s d of approximately 0.22 derived from the observed means and standard deviations of age among PTSD and non-PTSD groups, yielded a power of 0.290. This low power underscores the potential for a Type II error, suggesting the need for a larger sample size to reliably detect potential age-related differences in PTSD prevalence, if existent.

Our analysis indicated a significant association between the living environment and occurrence of PTSD. We observed that a higher proportion of patients with PTSD resided in rural areas (61 out of 86), compared to those without PTSD (44 out of 80), yielding a *p*-value of 0.033.

The Emergency County Hospital of Craiova is a main pillar in the evaluation of traumas in all areas of Oltenia. Therefore, this study also examined the distribution of patients with and without PTSD based on their county of residence. The patients were categorised into five groups based on the county of residence: Dolj, Gorj, Vâlcea, Olt, or Mehedinti. In assessing the geographical distribution of PTSD symptoms’ prevalence, significant variations were noted among different counties of residence. The data, substantiated by a *p*-value of less than 0.001, highlighted a pronounced disparity. Some counties reported a higher incidence of PTSD cases. This underscores the necessity to consider geographical variables in assessing and addressing PTSD, indicating that the location may indeed influence the disorder’s onset and progression.

Educational attainment, stratified into primary, secondary, and tertiary levels, was scrutinised in correlation with PTSD symptoms. The analysis revealed a nuanced distinction. The statistical significance of this disparity, corroborated by a *p*-value of 0.018, underscores a tangible correlation between education levels and PTSD onset. It highlights a pattern where the diversity in educational backgrounds is significantly less among PTSD patients, suggesting that educational attainment may influence vulnerability to, or resilience against, the disorder.

### 3.2. Injury Characteristics

This study also examined the association between PTSD and injury characteristics.

The patients were categorised into four unique groups based on the etiology of their hand trauma, specifically crush injuries, injuries caused by tools, injuries arising from traffic accidents, and injuries caused by glass shards. The tools included a grinder, circular saw, knife, cutter, chainsaw, electric scissors, and axe. The results of this study suggest the possible presence of differences in the distribution of causes of hand trauma among individuals with and without PTSD.

This study also examined the distribution of patients with and without PTSD based on the hand affected by the trauma. The patients were categorised into two groups based on the hand affected: right and left. The involvement of the left hand was predominantly observed. A statistical analysis was conducted to determine if there was a significant difference in the distribution of affected hands between the two groups. The results yielded a *p*-value of 0.010, which suggests that the hand affected by the trauma may play a role in the development of PTSD among hand trauma patients.

**Figure 1:**
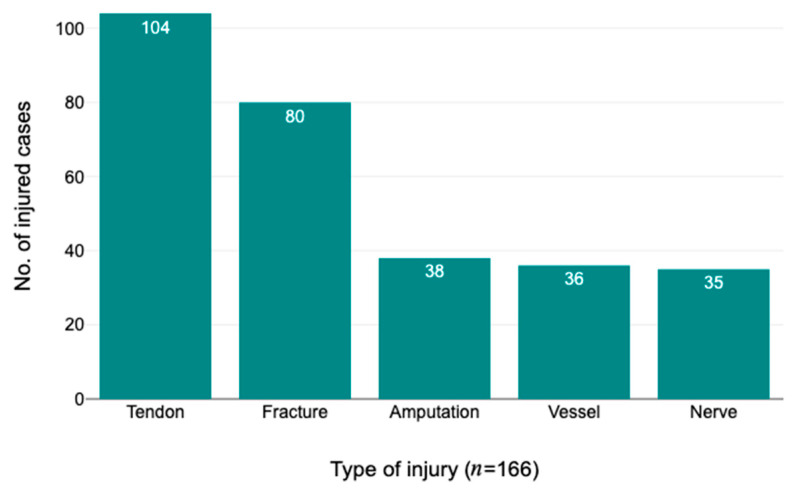
Type of injury.

The investigation also analysed the categorisation of patients according to the specific type of hand damage they had incurred (Figure 1).

The findings of this study offer valuable insights into the occurrence rates of several categories of hand injuries among the participants. The number of implicated tendons was quantified and afterwards utilised as metric data, which was subsequently compared between two distinct groups: those diagnosed with PTSD symptoms and individuals without PTSD. A two-tailed independent samples *t*-test was conducted to examine the statistical significance of the difference between the two groups and the results indicated a statistically significant difference, *p* = 0.015, with a 95% confidence interval ranging from 0.17 to 1.57.

We found a significant association between amputation and the diagnosis of PTSD, as evidenced by a calculated *p*-value of 0.03, indicating that there is a significantly higher likelihood for individuals with initial amputations to be diagnosed with PTSD symptoms compared to those without amputations. Based on the outcomes of the chi-square analysis, which indicated a statistically significant association between the presence or absence of a fracture and PTSD, it indicates a possible connection between these two variables (*p* = 0.003).

No significant connections were observed between patients with PTSD symptoms and the sections of nerves or blood vessels. Power analyses for the associations between PTSD and nervous or blood vessel lesions, using the observed chi-square values and Cohen’s d as effect size measures, resulted in powers of 0.340 and 0.390, respectively. These values indicate an underpowered study and a potential for Type II errors in these parameters, suggesting the need for a larger sample size to reliably detect potential correlations.

### 3.3. Postoperative Outcomes

All patients had surgical interventions based on the specific characteristics of their individual cases, including procedures such as osteosynthesis, arterial ligation, neuroraphy, and tenorrhaphy. This study investigated the postoperative outcomes of the participants (Table 2).

The patients were classified into two distinct groups according to their postoperative evolution: those with favourable outcomes and those with unfavourable outcomes. A statistical analysis was performed to assess whether there existed a statistically significant disparity in the distribution of postoperative outcomes between the two groups. The obtained data demonstrated a *p*-value that was found to be less than 0.001. The results of this study indicate that PTSD symptoms may be influenced by the recovery process of patients with hand trauma following surgery.

We also examined the need for amputation of necessity among patients with and without PTSD symptoms. A statistical analysis was conducted to determine if there was a significant difference in the occurrence of amputation of necessity between the two groups. The results yielded a statistically significant difference (*p* < 0.001) in the occurrence of amputation of necessity between patients with and without PTSD. These findings suggest that amputation of necessity during surgery or after may play a role in developing PTSD among hand trauma patients.

This study examined the occurrence of subsequent surgical intervention among patients with and without PTSD. The patients were categorised into two groups based on whether or not they required subsequent surgical intervention: yes and no. The results (*p* = 0.004) indicate that there is a statistically significant difference in the occurrence of subsequent surgical intervention between patients with and without PTSD, and subsequent surgical intervention may play a role in PTSD among hand trauma patients.

### 3.4. Family Support

**Figure 2:**
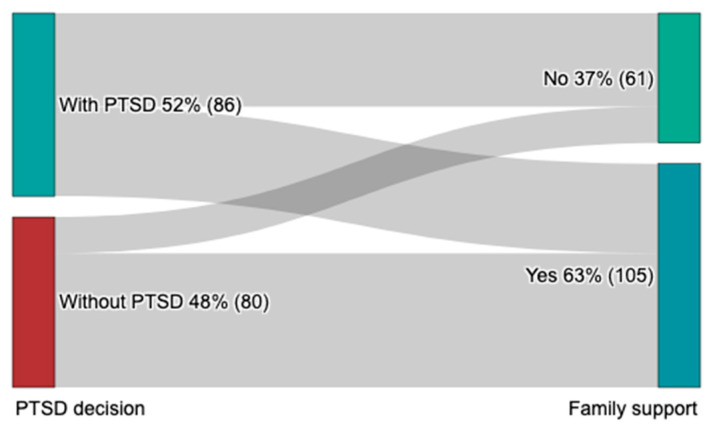
Family support and PTSD symptoms.

Family support (Figure 2) was identified to have a significant association with PTSD prevalence among hand trauma patients (*p* < 0.001). The data suggest a notable difference in PTSD occurrence based on the availability of family support.

## 4. Discussion

The current study examined factors that can predict the manifestation of PTSD symptoms in Romanian individuals who have recently had acute hand injuries.

The presence of psychological distress has a significant impact on the rehabilitation process of individuals with physical trauma and is closely linked to the probability of experiencing long-term disability, chronic pain, and a reduced overall sense of well-being [30,31]. The observed rise in psychological symptoms, as documented in this study, aligns with findings from previous research on musculoskeletal injuries and trauma survivors in general [31]. In light of the aforementioned points, it is crucial to underscore the necessity of implementing a holistic approach to healthcare for all individuals. The task of deriving significant quantitative findings from studies is complex due to the presence of diverse study methodologies, varying diagnostic instruments, and the utilisation of different cut-off values [32].

The finding that men in our study were significantly associated with PTSD post-hand trauma is indeed an observation that merits comprehensive discussion, especially in light of the prevailing literature that often associates PTSD more frequently with females. One pivotal aspect to consider is the prevalence of hand injuries among men, particularly owing to occupational exposure. Men are predominantly engaged in professions that are associated with a higher risk of hand injuries, such as manufacturing, construction, and manual labour [33]. These professions not only predispose them to a higher incidence of hand injuries but also expose them to traumatic events that could potentially lead to the development of PTSD.

The geographic disparity observed in the incidence of PTSD post-hand trauma underscores a complex interplay of socio-economic, cultural, and healthcare accessibility factors that vary significantly across different counties. In our study, the data exhibited a notable variance in PTSD prevalence among hand trauma patients from different counties, pointing towards an intricate matrix of locational determinants that influence the psychological aftermath of physical trauma. In the context of our study, patients living in counties with limited access to specialised healthcare, reduced socioeconomic status, and pronounced stigmas associated with mental health may be at a greater risk of developing and persisting PTSD symptoms. These counties may lack the necessary infrastructure or social support mechanisms to facilitate early identification, intervention, and comprehensive management of hand-trauma and PTSD symptoms.

The findings of our investigation were consistent with the existing literature regarding the correlation between patients’ degree of education and socioeconomic position. Lower levels of education are linked to a higher susceptibility to trauma and PTSD and have fewer cognitive and psychological resources to handle such experiences [34]. Moreover, lower levels of education are often associated with less money, which can make it harder for people to obtain the healthcare and support they need [35]. In contrast, individuals with higher education frequently exhibit improved coping skills and psychological resilience, which can help alleviate the severity of PTSD symptoms. These individuals are also more likely to have less physically demanding occupations, resulting in a lower risk of hand injuries [36]. Individuals with higher education and socioeconomic status have better access to medical and psychological care, leading to improved outcomes after hand trauma.

Given the profound significance attributed to hands, it is comprehensible that individuals who have experienced hand trauma may exhibit heightened vulnerability to psychological suffering. The existing societal stigma around disability results in the potential for feelings of insecurity, ineptitude, and dependency when faced with a nonfunctional hand. These reactions are understandably unpleasant from a psychological standpoint [37]. The recollection of the injury and the visual perception of the hand can elicit an effective response and give rise to psychological anguish [32].

While a considerable proportion of individuals exhibit spontaneous recovery from the psychological consequences of trauma, a subgroup endures symptoms of PTSD, thereby emphasising the intricate range of post-traumatic reactions and emphasising the typicality of natural recovery [38,39,40]. The findings of this study align with previous research [24] and indicate that it may be appropriate to consider screening for psychological symptoms at the 3-month mark in order to identify hand trauma patients who may benefit from additional treatment following their traumatic event.

Our results suggest that family support plays a role in the development of PTSD among hand trauma patients. The varied nature of potential reasons for the influence of family support on the risks of developing PTSD following hand trauma surgery is evident. The provision of familial support can offer individuals undergoing surgery emotional solace, comprehension, and a feeling of inclusion, thereby aiding in their ability to manage the distressing encounters linked to their surgical procedures [41]. In addition, familial support can offer tangible aid throughout the recuperation phase, potentially facilitating a more seamless and less burdensome postoperative period. This support includes aid with performing routine activities, offering transportation services for medical visits, and facilitating the management of medication. The implementation of practical forms of support has the ability to lessen the load on patients and mitigate stress, hence potentially diminishing the likelihood of developing PTSD [41].

Previous research has consistently identified substantial social support post-injury as a mitigating factor for the development of PTSD symptoms [35]. In contrast, individual attributes including being young, female, and having lower educational attainment, coupled with environmental factors, like low socioeconomic status and severe trauma exposure, have been associated with an increased PTSD risk [34,36]. Furthermore, studies focusing on male subjects have reported amplified effects in cases involving younger age at trauma exposure, poorer socioeconomic conditions, and minority racial status, compared to studies concentrating on female subjects [35].

A significant prevalence of PTSD symptoms has been noted among individuals who have experienced trauma specifically affecting their right hand. There could be several potential explanations for the observed association between hand laterality and PTSD diagnosis. There is a possibility is that the dominant hand, which is typically the right hand for most individuals, may play a more significant role in daily activities and tasks. Trauma to the dominant hand could, therefore, have a greater impact on an individual’s ability to perform daily tasks and may result in increased stress and anxiety, potentially contributing to the development of PTSD symptoms.

There are a wide variety of probable causes for the increased risk of developing PTSD, regarding postoperative outcomes. One potential explanation is that the psychological distress resulting from the initial surgical intervention, in conjunction with the subsequent requirement for supplementary surgical procedures, may contribute to increased levels of stress and anxiety. The extended exposure to medical treatments has the potential to contribute to the manifestation of symptoms associated with PTSD [42]. Moreover, the heightened susceptibility to PTSD may be attributed to the combined impact of numerous surgical procedures, including both physical and emotional strain, as well as probable difficulties and setbacks encountered during the recuperation process. Elevated levels of distress and the emergence of symptoms associated with PTSD might be attributed to various factors, including heightened pain, prolonged hospitalisation, and lengthy durations of impairment [43]. The implementation of routine screenings to assess symptoms of PTSD, the early identification of people who are at a higher risk for developing PTSD, and the provision of psychological support services have been shown to be effective strategies in minimising the adverse effects of PTSD on patients’ well-being, recovery process, and overall quality of life [42].

Furthermore, it is crucial to examine the ramifications of cPTSD in conjunction with the investigation of PTSD, subsequent to hand trauma. Hand trauma, which can occur due to interpersonal violence, accidents, or catastrophic occurrences, encompasses not only physical harm but also significant psychological distress [44]. The aforementioned traumatic experiences have the potential to occur repeatedly or over an extended period of time, resulting in a complex interaction of reactions similar to cPTSD. This disorder is characterised by difficulty in regulating emotions, a negative perception of oneself, and challenges in interpersonal relationships [45].

Individuals who have had hand trauma, particularly those who have undergone several surgeries or extended rehabilitation, may have symptoms of cPTSD that go beyond the traditional symptomatology associated with PTSD. The comprehension of how cPTSD presents itself might contribute to the development of specific interventions, emphasising the importance of individualised strategies in psychological evaluations and treatments [46].

The transition from PTSD to cPTSD highlights the underlying gravity and prolonged duration of the psychological consequences associated with hand trauma. The aforementioned alteration carries significant implications, as it is associated with a range of detrimental consequences, such as an elevated likelihood of engaging in suicidal behaviour, heightened vulnerability to experiencing psychosis, and an augmented inclination towards substance misuse [47]. These results highlight the importance of adopting a proactive, well-informed, and comprehensive strategy to provide psychological care for patients with hand trauma, extending beyond the initial period following the injury. This underscores the necessity for implementing long-term mental health initiatives.

A considerable number of patients in our study, under the impact of prevailing society conventions, failed to recognise the necessity of undergoing psychological evaluation or seeking therapeutic help. The participants refrained from aligning themselves with the negative connotations surrounding mental health difficulties, leading them to withdraw from the later stages of the research and not be included.

The aforementioned dynamics had a dual effect on our sample size, while also shedding light on the complex relationship between societal standards, individual views, and the expression and recognition of symptoms associated with PTSD.

Our findings corroborate the documented prevalence of PTSD following hand trauma and elevate the discourse by elucidating intricate relationships. We unveil the nuanced interplay of socio-demographic variables, distinct trauma types, and their cumulative impact on PTSD symptomatology. This granular analysis aims to augment the prevailing understanding, offering actionable insights for tailored, patient-centric interventions.

By delineating specific risk constellations and their differential impacts, our study aspires to equip clinicians and caregivers with refined tools for anticipatory guidance, timely interventions, and enhanced support for afflicted individuals.

In this light, our study transcends the descriptive scope, offering predictive insights and actionable intelligence to enhance the precision and efficacy of PTSD management post-hand trauma. It represents a step forward in translating empirical data into clinical practice, aligning interventions with individual needs for optimised recovery outcomes.

Our study serves as a preliminary investigation into the factors influencing PTSD development. In future studies, we plan to build upon our current findings by enlarging the sample, employing multivariate techniques to examine the interplay between multiple variables, and providing a deeper understanding of the complex factors contributing to PTSD development.

## 5. Conclusions

Our investigation elucidates a complex interplay of factors contributing to the emergence of PTSD following hand trauma, underscoring a nuanced landscape of vulnerabilities and triggers. Gender, residential environment, geographical location, educational attainment, occupational exposure, surgical aftermath, familial support, and the nature and locus of the trauma surfaced as integral elements in this intricate matrix.

The prevalence of PTSD emanated predominantly from traumas associated with crushing or cutting incidents, with bones and tendons being the most impacted structures. In contrast, nerves and vessels did not exhibit a significant correlation with PTSD manifestation, but it is important to note that these findings are based on a relatively small sample size and further research with a larger sample is needed to draw more definitive conclusions. These insights suggest a compound interrelationship of individual attributes, trauma specifics, and post-traumatic responses delineating the PTSD trajectory.

Recognising the ubiquity of psychological distress post-traumatic injury, this study amplifies the call for systematic psychological assessments. We advocate for the incorporation of targeted screenings at the three-month juncture post-injury, a critical window where enduring psychological afflictions can be identified and addressed. Such evaluations can be seamlessly integrated into routine follow-ups or conducted remotely, fostering an environment where psychological well-being is accorded equal precedence.

The insights gleaned from this study do not merely iterate observations but contribute substantively to refining the clinical approach to post-hand trauma care. We illustrate a detailed landscape of PTSD emergence, fostering anticipatory interventions and personalised care protocols, thereby transitioning from a one-size-fits-all model to a patient-centric care paradigm.

While our findings illuminate previously obscured facets of PTSD post-hand trauma, we acknowledge the necessity for expansive research. Studies encapsulating diverse samples, deploying an array of evaluative instruments, and adopting longitudinal designs are paramount to delineate, with precision, the multifarious dimensions of PTSD’s psychological aftermath.

In essence, this research stands not as a conclusion but as a pivotal juncture, propelling the discourse into arenas marked by precision, individualisation, and comprehensive care. Every insight gleaned paves the path for nuanced understanding, refined interventions, and holistic healing, marking not just physical recuperation but psychological restoration as concurrent imperatives in the aftermath of hand trauma.

## Figures and Tables

**Table 1:** The demographic characteristics of the participants.

Characteristics	Probable PTSD (*n* = 86)	without PTSD (*n* = 80)	*p* Value
Gender			0.004
Female	19	5	
Male	67	75	
Age			>0.05
18–29	18	15	
30–49	36	44	
50–70	32	21	
Environment			0.033
Rural	61	44	
Urban	25	36	
County			<0.001
Dolj	45	61	
Gorj	13	7	
Valcea	14	4	
Olt	4	8	
Mehedinti	10	0	
Education			0.018
Primary	3	3	
Secondary	63	42	
Higher	20	35	
Occupation			<0.001
Social aid	5	0	
Unemployed	32	11	
Retired	20	6	
Employed	25	54	
Student	4	9	

**Table 2:** The postoperative outcomes of the participants.

Characteristics	with PTSD (*n* = 86)	without PTSD (*n* = 80)	*p* Value
Postoperative evolution			<0.001
Unfavourable	26	4	
Favourable	60	76	
Amputation of necessity			<0.001
No	62	80	
Yes	24	0	
Subsequent surgical intervention			0.004
No	61	70	
Yes	25	10	

## Data Availability

The data presented in this study are available on request from the corresponding author. The data are not publicly available due to privacy and ethical considerations to safeguard the confidentiality of the participants’ information.
